# Towards a Unified Understanding of Event-Related Changes in the EEG: The Firefly Model of Synchronization through Cross-Frequency Phase Modulation

**DOI:** 10.1371/journal.pone.0045630

**Published:** 2012-09-25

**Authors:** Adrian P. Burgess

**Affiliations:** Aston Brain Centre, Aston University, Birmingham, United Kingdom; University of British Columbia, Canada

## Abstract

Although event-related potentials (ERPs) are widely used to study sensory, perceptual and cognitive processes, it remains unknown whether they are phase-locked signals superimposed upon the ongoing electroencephalogram (EEG) or result from phase-alignment of the EEG. Previous attempts to discriminate between these hypotheses have been unsuccessful but here a new test is presented based on the prediction that ERPs generated by phase-alignment will be associated with event-related changes in frequency whereas evoked-ERPs will not. Using empirical mode decomposition (EMD), which allows measurement of narrow-band changes in the EEG without predefining frequency bands, evidence was found for transient frequency slowing in recognition memory ERPs but not in simulated data derived from the evoked model. Furthermore, the timing of phase-alignment was frequency dependent with the earliest alignment occurring at high frequencies. Based on these findings, the Firefly model was developed, which proposes that both evoked and induced power changes derive from frequency-dependent phase-alignment of the ongoing EEG. Simulated data derived from the Firefly model provided a close match with empirical data and the model was able to account for i) the shape and timing of ERPs at different scalp sites, ii) the event-related desynchronization in alpha and synchronization in theta, and iii) changes in the power density spectrum from the pre-stimulus baseline to the post-stimulus period. The Firefly Model, therefore, provides not only a unifying account of event-related changes in the EEG but also a possible mechanism for cross-frequency information processing.

## Introduction

The electroencephalogram (EEG) has made a very significant contribution towards our understanding of human sensory and cognitive processes, and foremost in this regard have been studies investigating changes to the EEG resulting from the presentation of a stimulus or the making of a movement. Event-related potentials (ERPs) are a widely used tool for this purpose but despite their undoubted success, there remains a fundamental uncertainty as to what they are and how they are generated. The core uncertainty is whether the ERP is an evoked signal (i.e. a signal super-imposed upon and independent of the ongoing EEG), a phase alignment of the ongoing EEG, or some combination of the two.

This is no arcane debate as the nature of the ERP goes to the heart of numerous central issues in the field. If the ERP is an evoked signal, it makes perfect sense to measure the amplitude and latency of ERP maxima and minima and to try and localise the sources of ERP components and identify their functions. If instead the ERP emerges from a phase alignment of the ongoing EEG, then the maxima and minima of the ERP may have no real significance and the idea that ERP components have clearly identifiable sources and functions might prove illusory.

### Induced and Evoked Responses

The controversy over the nature of the ERP is significantly complicated by the fact that there are two classes of event-related change in the EEG that invariably accompany each other but which appear to be largely independent [1]: induced and evoked responses. The physiological mechanisms that generate evoked and induced event-related changes in the EEG have not been fully characterised but a clear conceptual exposition of the difference is provided by Pfurtscheller & Lopes da Silva [2]. Essentially, induced changes are power changes in the ongoing EEG whereas evoked changes are event-related transients generated by the temporary synchronization of networks of neurones and are independent of the ongoing EEG.

As induced changes involve event-related power changes in the ongoing EEG, they are time-locked to the event but do not show a consistent phase response. An example of this is the classic Berger effect, whereby the alpha rhythm, an EEG oscillation of around 10 Hz seen over the posterior scalp during waking, is attenuated by opening the eyes. The Berger effect is time-locked in that it occurs at the time the eyes are opened but it is not phase-locked because the alpha rhythm is attenuated regardless of what phase the alpha rhythm happens to be in at the time the eyes are closed. Induced changes go under a variety of names including event-related synchronization (ERS) and event-related desynchronization (ERD) depending upon whether the power increases (synchronization) or decreases (desynchronization) [2] and more generally as ‘event-related spectral perturbations’ [3], [4].

If, as is widely assumed, evoked changes are responses generated by the temporary synchronization of networks of neurones that are independent of the ongoing EEG, then they would be both phase-locked and time-locked to the event. Phase-locked is a term best suited to describing oscillatory phenomena and means that the event induces a consistent phase in the ongoing oscillation. The term is curiously inappropriate when referring to changes that are non-oscillatory but the terminology is well-established and will be maintained here. Phase-locked in this context simply means that the generated field has a consistent shape and time course from one trial to the next.

**Figure 1:**
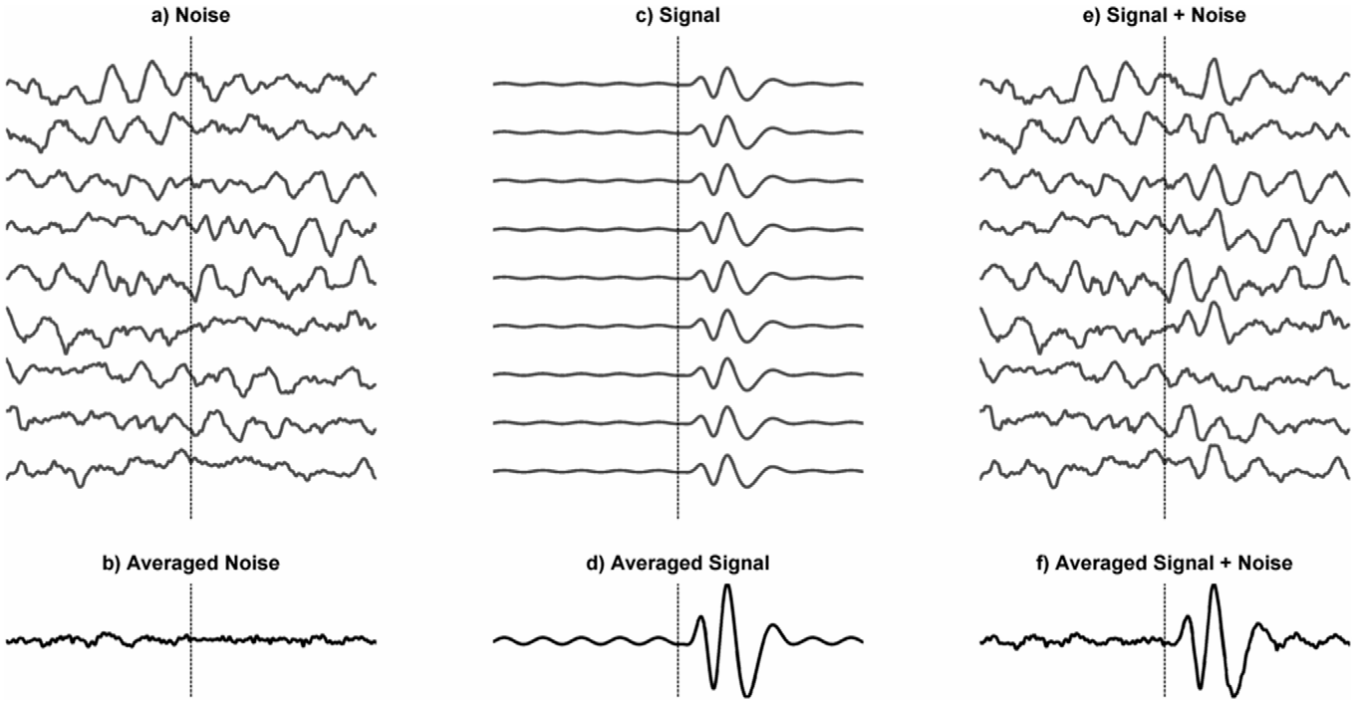
Schematic Representation of the Evoked Model of ERP Generation. In this model the ERP is conceptualised as being an evoked signal superimposed upon, and independent of, the background EEG. Panel a) represents multiple segments of the ongoing EEG in which an event occurred such as the presentation of a stimulus or initiation of a movement. The time of the event is indicated by the vertical dotted line. In this model the ongoing EEG is treated as noise. Panel b) is the average of the multiple segments of EEG and shows that the oscillations tend to cancel out producing a flat line. Panel c) shows the signal evoked by the event which is consistent across all segments and d) represents the average of those evoked signals. Panel e) shows the recorded EEG which is the sum of a) and c). Finally, panel f) shows that the mean of the segments of recorded EEG from e) provides an accurate reconstruction of the evoked signal.

### EEG Frequency Bands

At this stage, it is worth considering the frequency distribution of the oscillations observed in the spontaneous EEG. By convention, the waking EEG is divided into five distinct frequency bands: delta (0.5–4 Hz), theta (4–8 Hz), alpha (8–13 Hz) beta (13–30 Hz) and gamma (>30 Hz). Each of these bands may be further subdivided so, for example, the beta band is often subdivided into beta1 (13–20 Hz) and beta2 (20–30 Hz) and so on. With the exception of alpha, these frequency bands are not associated with identifiable peaks in the power spectrum. Indeed, the distribution of power in the EEG is approximately proportional to 1/frequency, except for the alpha peak (∼10 Hz) which is most prominent at rest with the eyes closed.

Induced responses involve power changes to the amplitude of the ongoing EEG but the direction of change depends upon the frequency band in question. Presentation of a visual stimulus, for example, will typically result in a decrease in the amplitude of alpha (i.e. desynchronization) but an increase in amplitude in theta (i.e. synchronization) [5], [6], Why cortical oscillations in some frequency ranges synchronize whereas others desynchronize has never been adequately explained before but a possible answer to this question will be provided later.

The observation that the type of induced power changes seen in the EEG are frequency dependent and are differentially responsive to experimental manipulation, has often been interpreted to mean that different EEG frequency bands have specific functions (see, for example [6]). However, although the search for specific functions for specific frequency bands has a long history it has not been overburdened by success. Nevertheless, if distinct frequency bands exist it makes sense to ask how many there are and what frequency ranges they occupy. The answers to these questions remain uncertain but evidence from both human and *in vitro* studies point to as many as 8 distinct frequency bands between 1.5 and 80 Hz [7], [8]. It has also been noted that the spacing of the frequency bands follows a mathematical pattern that is optimal for minimizing cross-frequency interference between them [7]–[9]. If so, it suggests that the cortex may use frequency-division multiplexing with each frequency band conveying its own independent stream of information. Complete informational encapsulation, however, would be of little use unless there were some additional mechanism for communicating between frequencies [10] and there is good evidence that such communication exists [11]–[13] A novel mechanism for how cross-frequency communication may be achieved will be proposed later.

### What is an Event-related Potential?

For nearly forty years, two competing mechanisms have been proposed to account for the generation of ERPs: the evoked model and the phase-alignment model. As will become clear, neither model is entirely satisfactory nor are they mutually exclusive.

The evoked model of the ERP is illustrated in Figure 1. In this model, the ERP is an evoked signal superimposed upon the ongoing EEG and averaging across trials is simply a means of increasing the signal to noise ratio; the evoked components (signal) which are phase-locked are kept whilst the ongoing EEG (noise), which is not phase-locked’* cancels out. If the evoked model is correct, it has many useful properties. It is, for example, the underlying assumption that justifies the search for the functional significance of ERP components and their anatomical substrates.

One limitation of the evoked model is that it provides no satisfactory explanation for the characteristic shape of ERPs. The evoked model explains the ERP as being the summation of multiple, evoked responses generated from different sources with different time courses. In principle, such a model can explain the time course of any possible ERP. Because it can explain everything, however, in an important sense it explains nothing. Sensory and cognitive ERPs, for example, typically have the appearance of an amplitude modulated ‘down-chirp’. A down-chirp is a signal in which the frequency decreases with time and, in the case of sensory and cognitive ERPs, the amplitude is modulated such that it is inversely proportional to frequency. This pattern suggests that ERPs have an underlying structure in the frequency domain and it is a pattern that cannot easily be accounted for by the evoked model.

**Figure 2:**
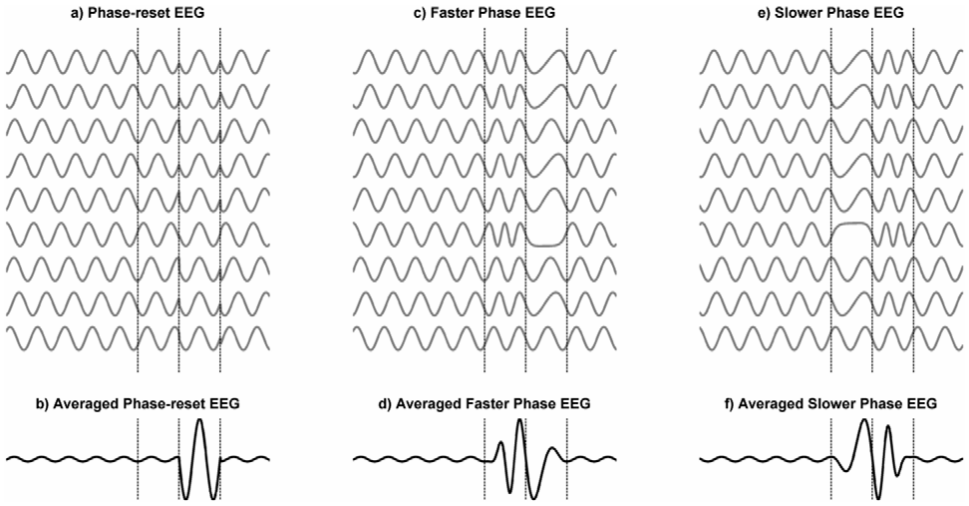
Schematic Representation of three variants of the Phase-alignment Model. In this model, ERPs are considered to be a re-organisation of the ongoing EEG in response to an event (e.g. the presentation of a stimulus or initiation of a movement) and there is no evoked signal. Panel a) represents the phase reset model and shows multiple segments of the ongoing EEG in which an event occurred such as the presentation of a stimulus or initiation of a movement. The time of the event is indicated by the first vertical dotted line. In each case, the event induces an instantaneous shift in the phase of the ongoing EEG. Panel b) shows the mean of the segments in a) and shows an ERP-like response. Panel c) is similar to a) except that the change of phase is not instantaneous but gradual and the phases come into alignment by the time of the 2^nd^ dotted line by increasing the rate of change of phase. The oscillations then slow down and return to their initial phase by the time of the 3^rd^ dotted line. Panel d) shows the average of the oscillations in c). Panels e) and f) are identical to c) and d) except that the initial phase alignment is achieved through slowing down and the return to baseline phase by speeding up. Note that the shape of the ERPs in panels b), d), and f) differ.

The initial challenge to the evoked model came from Sayers, Beagley, & Henshall [14] who argued that if ERPs are generated by evoked signals superimposed on the ongoing EEG, then the power in the EEG signal post-stimulus should be higher than that in the pre-stimulus period. Having measured pre- and post-stimulus power, and finding no evidence of an increase in power, Sayers et al.[14] argued that the evoked model was not sustainable and proposed instead that the ERP emerged from a phase re-organisation of the ongoing EEG. As the EEG consists of oscillations across a range of frequencies, summations of the signal will average to zero because the positive and negative peaks will cancel out. However, in the phase alignment model, the presentation of a stimulus causes the oscillations to shift phase in such a way that positive and negative peaks align. In such circumstances, the positive and negative peaks will not cancel out but will summate to form an ERP (Figure 2a). In this model, both phase-locked and non-phase-locked changes in the EEG arise from modifications of the underlying ongoing oscillations and there is no need to invoke an evoked signal at all. Further support for this idea comes from an extensive body of evidence showing a link between the magnitude of ERP components and the power of the EEG in the pre-stimulus period [15].

One limitation of the original phase re-alignment model was that it was never fully specified [14] In fact, the model illustrated in Figure 2a), known as the phase re-setting model because at some point post stimulus onset the oscillations in the EEG are abruptly forced to the same phase, is only one of a number of models that attempt to explain the ERP in terms of re-organisation of the phases of the ongoing EEG. The phase-resetting model assumes an instantaneous, or at least a very rapid, shift in phase. If instead, there is a progressive shift over time, then phase alignment can be achieved by the change of phase either increasing or decreasing (Figure 2b&c), or some combination of the two. Other phase alignment models have been proposed [16] and no doubt others could be conceived.

One immediate consequence of all the variants of the phase-alignment model is that the peaks and troughs of the ERP are simply artefacts of the phase-re-organisation of the oscillatory components of the EEG. This means that instead of having a localised source, the peaks and troughs emerge from the phase alignment of neural oscillations across what is most likely to be a large area of cortex. Whereas the evoked model generates an easily visualisable mental picture in which a localised area of cortex increases its activity as it performs a specific function and in consequence generates an ERP peak (e.g. face processing and the N170), the phase-alignment model provides no such comforting picture. There is no change in power, just a phase-alignment and, worse still, rather than occurring in a well-defined location, there is good reason to think that this phase-alignment spreads across the cortex like a travelling wave [17]–[19]).

There are two problems with all of the phase-alignment models that are usually overlooked. Illustrations like that in Figure 2 invariably show phase re-organisation in oscillations with a single initial frequency and when averaged over many trials, they do generate an event-related response. The first problem is that the model predicts that there is phase synchronization between trials but not within a single trial. This means that the ERP does not exist in any individual trial but is an emergent property of multiple trials.

The second problem, which is closely allied to the first, is how does the model work with oscillations spanning a broad range of frequencies? If each frequency component synchronizes at the same phase and time, the average does not look ERP-like at all. Alternatively, if the frequencies are aligned to random phases, then the oscillations cancel out and no ERP is formed. Clearly, if these models are to be made to work, then either the phase-alignment must either be restricted to a narrow frequency band or there must be some systematic arrangement of phases across frequencies. One clue for what this arrangement might be comes from the amplitude-modulated chirp-like shape of sensory and cognitive ERPs. This chirp-like structure suggests that phase alignment is frequency dependent with lower frequencies taking longer to align than higher frequencies. The amplitude modulation suggests that power is inversely proportional to frequency, just like the ongoing EEG which is surely no coincidence.

The solution to the second problem, if it can be found, may answer the first problem too. If there is some systematic phase alignment across frequencies, then the issue of phase alignment across trials does not arise; the alignment is within-trial. It also means that the ERP is no longer an emergent property of multiple trials but can exist in individual trials as well. This will come as some relief, if not surprise, to those who study single trial ERPs.

Although the phase alignment model of ERPs has never achieved the widespread acceptance of the evoked model it has never gone away and interest in it has grown, particularly over the last decade. This renaissance of interest is perhaps unsurprising because the phase alignment model has a number of attractive features. Most importantly, it offers a unifying perspective on event-related changes in the EEG in several ways. First, it unifies the study of the EEG which for several decades has followed two largely independent paths. One path has focused on the study of the ongoing EEG and emphasised the clinical and the physiological. The other path has focused on ERPs, largely from a psychological perspective and considered the EEG to be noise. All too often these paths have been treated as independent lines of investigation, often conducted by separate groups of researchers. If the phase-alignment model is correct, this distinction would no longer be sustainable. Second, the phase-alignment model reconnects ERP studies with the broader field of neuroscience, particularly with electrophysiology where the importance of cortical oscillations and how they are generated has long been an important area of study. Finally, the phase-alignment interpretation of ERPs makes potentially important links with the study of complex systems (e.g. synergetics, [20]) where phase relationships have been found to be so important.

Although the evoked and phase-alignment models have dominated discussions about the nature of the ERP for several decades, they are no longer the only candidate mechanisms. Recently, a completely new mechanism based on asymmetric amplitude modulation (AAM) of the alpha rhythm, has been postulated [21], [22]. The AAM model has a number of attractive features. First, it is based on empirical observation, derived from ICA decomposition of the ongoing EEG that the peaks and troughs of the alpha rhythm fluctuate asymmetrically. Second, the model assumes simple amplitude modulation of the underlying EEG. Third, it provides a unifying account of both evoked and induced changes in the EEG. However, the model does not provide an explicit explanation for the shape and timing of either the induced or evoked changes in the EEG.

### Tests of the Evoked Model of ERPs

Many attempts have been made to determine whether the evoked model or one of the variants of the phase-re-setting model, best fits with observation. It is beyond the scope of this paper to go into this debate in detail but good reviews are available for the interested reader [23], [24]. However, it is useful to outline briefly the main approaches that have been taken to test the evoked and phase-alignment models before proceeding to propose an alternative test of these hypotheses.

#### The power test

Following Sayers et al [14] the most direct method to test the evoked and phase-alignment models is to compare the power in the pre- and post-stimulus periods. Under the evoked model, power should be greater in the post-stimulus than the pre-stimulus period because the evoked signal is superimposed upon the ongoing EEG; under the phase-alignment model, the powers should be equal. Unfortunately, this approach ignores power changes that are not stimulus phase-locked (i.e. induced power changes) and for this reason, the method fails.

The power method could easily be redeemed if only evoked and induced power changes could be disentangled. A method to decompose the EEG signal into phase-locked and non-phase-locked components has been proposed [25] but, unfortunately, this does not resolve the problem because it only works if the evoked signal is exactly the same on every trial [26]. Such precision in any physiological system cannot be safely assumed and without any alternative means of disentangling the induced and evoked components, the power method must be abandoned.

#### The phase concentration test

One clear prediction of the phase-alignment models is that there should be an increase in phase-synchrony or an increased concentration of phase in the post-stimulus period that should be readily measureable. Some studies have indeed demonstrated such an increase [27].but unfortunately this evidence does not discriminate between the evoked and phase resetting models [23] because the components of an evoked signal have exactly the same phase across trials which will contribute to any measure of phase synchrony that is used.

#### The pre-stimulus prediction test

If the phase alignment models are correct, there should be reliable dependencies between the ongoing EEG in the pre-stimulus period and the ERP. Many different measures have been used but recently there has been much interest in phase at the time of stimulus onset. Single trials are sorted by their phase and the ERPs calculated on subsections of these trials (e.g. negative phase vs. positive phase). Under the evoked model, it seems reasonable to assume that baseline phase should be irrelevant and the ERPs from the positive and negative phase trials should be the same. In fact, this turns out not to be the case because the pre-stimulus phase leaks into the post stimulus period [16]. This is also one case where the variants of the phase-alignment models give different predictions [16]. For example, the phase-resetting model makes the same prediction as the evoked model because the instantaneous re-set of phase means that there will be no association between phase in the pre- and post-stimulus periods. However, if instead of instantaneous phase resetting, the phases align over a short period of time, then a different outcome can be expected. This can happen in one of two ways. First, the change of phase over time is constant which means that the oscillations will align at a time dependent upon their starting phase. Second, the change of phase over time is variable, but the oscillations re-align at the same time point post-stimulus onset. In either case, the ERP generated will depend upon the initial phase at stimulus onset.

One problem with this approach is the difficulty in measuring phase. Phase, is not defined for broadband signals [28] so any measurement of phase necessitates some sort of bandpass filtering of the EEG. No filter is perfect and whichever method is used inevitably results in some degree of smearing in both the time and frequency domains. The result is that any filtering technique inevitably introduces dependencies between phase measurements at near time points. This problem can be alleviated by increasing the temporal precision of the filter only but only at the cost of reducing its frequency precision and this introduces other possible artefacts.

There is, however, a more fundamental problem with this approach. Under the evoked model, averaging segments of EEG reveals the ERP because the ongoing oscillations (i.e. noise) cancel out. However, the ongoing oscillations only cancel out if their phases are random. If the segments are systematically sorted by phase, then they are no longer random and don’t cancel out. The result is that ERPs generated from subsamples of phase-sorted segments of EEG will not, in general, be equal. Similar arguments apply to amplitude sorted trials.

### A New Test of the Evoked Model of ERPs

The debate between the evoked and phase alignment models remains unresolved but one prediction of the phase alignment models has not yet been tested: the frequency prediction. If, as the phase-alignment models assert, phases synchronize post-stimulus over a short period of time, then there must be a concomitant change in frequency. The reason for this is that phase alignment requires a change of phase and frequency is the change in phase over time (i.e. the 1^st^ derivative of phase). In the case of the evoked model, the change in phase is instantaneous which means that the frequency will transiently be infinite. In practice, one might see a transient peak in frequency (positive or negative) although this might be lost in any filtering or averaging that might be required. If however, phase alignment results from the change of phase over time slowing or speeding up, measureable changes in frequency should occur. As phase alignment is only transitory, it follows that once alignment has been achieved, further changes in phase (and hence frequency) must follow. If this did not happen, the oscillations would remain synchronized. Our expectation is that if the frequency of an oscillation slows down to align, it will speed up afterwards to return to its baseline frequency, or vice versa. Phase alignment, therefore, can be viewed as resulting from a transient deviation in the frequency of oscillation. However, an overall change in frequency will only be detected if increases in frequency predominate over decreases or vice versa. If increases and decreases occur equally often, they will cancel out, the overall change will be zero and no frequency change will be detected.

Although the phase-alignment models make clear predictions about the change in frequency of the EEG post-stimulus onset, there remains the problem of what we mean by frequency because for broadband signals, like the EEG, frequency is not defined [28]. What is needed is a method for decomposing the EEG into narrow-band components where frequency can be meaningfully defined. Many methods for analysing the EEG exist but the methods most widely used (e.g. FFT, wavelets and digital filters) are unsuitable for this purpose as they all require that frequency bands are pre-defined and constant. One suitable method, however, is empirical mode decomposition.

#### Empirical mode decomposition

**Figure 3:**
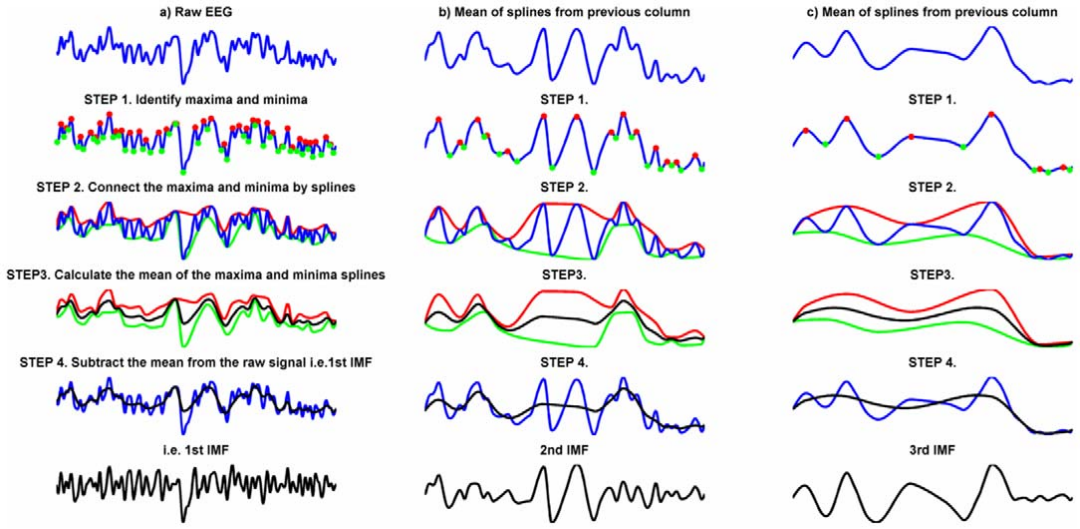
Schematic Representation of Empirical Mode Decomposition (EMD). Panel a) shows the steps taken to produce the 1^st^ intrinsic mode function (IMF). Step 1 involves identifying local maxima (red dots) and minima (green dots) of the signal. Step two involves linking the maxima together (red line), interpolating intervening points by a spline-fitted curve. The same is done for the minima (green line). In Step 3, the mean of the maxima and minima splines is calculated (black line) and subtracted from the original signal. The residual is the 1^st^ intrinsic mode function (black line). Panels b) and c) show the mean spline being subjected to Steps 1–4 to extract the 2^nd^ and 3^rd^ IMFs respectively. The process is repeated until the mean spline is monotonic and no more IMFs can be extracted.

EMD is a data driven method for decomposing a waveform into components, called Intrinsic Mode Functions (IMFs) that makes minimal assumptions about the nature of the signal and is suitable for non-stationary and nonlinear signals [29]. IMFs are comparable to harmonic functions in FFT but more general. Both are oscillations with zero mean derived from the decomposition of a signal that when summed together reconstitute the original signal. However, whereas harmonic functions have constant frequency and amplitude, the frequency and amplitude of IMFs may vary over time. EMD is described schematically in Figure 3. The highest and lowest IMFs are limited, but not defined by, the sampling rate and the length of the signal respectively. One cannot extract an IMF with a frequency higher than the Nyquist frequency, nor one with a longer period than the signal length. Notwithstanding these limits, neither the sampling rate nor the segment length is critical for defining the IMFs. Once the IMFs have been extracted, the Hilbert Transform can be used to estimate the instantaneous amplitude, phase and frequency of each. Simulations suggest that signal decomposition using EMD is typically superior to other methods such as wavelets [29] including use with EEG data [30]. For our purposes, however, the key advantage of EMD over other approaches is that it allows us to measure changes in EEG frequency without predefining frequency bands.

### Aims

It is proposed that the evoked and phase-alignment models of the ERP can be discriminated on the basis of event-related changes in EEG frequency. Specifically, if the phase-alignment model is correct, there should be a change in EEG frequency in the immediate post-stimulus period whereas if the evoked model is correct, no such frequency change should be seen. The first aim of this paper is to apply this test to EEG data collected from participants performing a standard cognitive task. The second aim is to use the results from this study to develop a new model of event-related changes in the EEG, the Firefly Model (presented in *Models*), that can account for the nature of both the evoked and induced changes in the EEG and which shows that they can be understood as different aspects of a single process.

## Materials and Methods

### Empirical Study

#### Participants

Participants were 20 healthy young adults (8 women) recruited through advertisement with a mean age of 26.0 (s.d.  = 5.6; range 19–41 years). Written informed consent was obtained from all subjects and the experiment was conducted as approved by the Riverside Research Ethics Committee. All investigations were conduction according to the principles expressed in the Declaration of Helsinki and data were analysed anonymously.

#### Procedure

**Figure 4:**
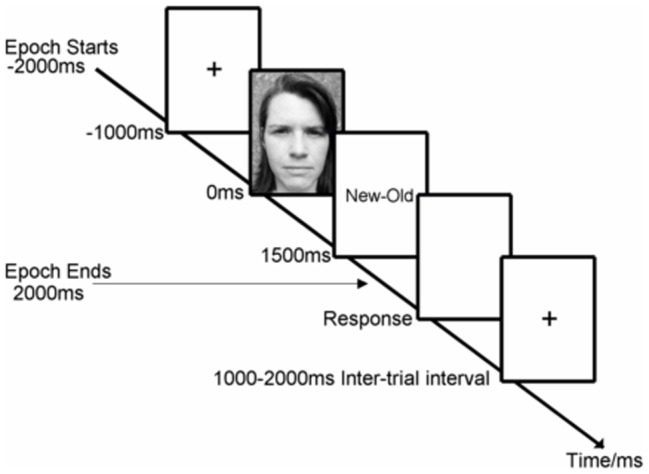
The sequence of events for the Face Recognition Memory Task.

EEG was recorded from participants as they performed a continuous recognition test for faces [31]. The test consisted of 90 trials each divided into three phases: a baseline cue, a memory stimulus, and a response cue. The sequence of events for each trial is shown in Figure 4.

The baseline cue consisted of a blank screen with a central fixation point. The stimuli were 50 faces from the Stirling University Psychological Image Collection (http:/pics.psych.stir.ac.uk) consisting of black and white photographs (approximately 5.0 cm wide by 6.5 cm high) of the head and shoulders of men and women (25 of each), with neutral emotional expressions, facing directly toward the participant. The faces were presented at the centre of a computer screen situated approximately 1.5 meters from the participant with the centre of the screen at eye level. The test included 40 stimuli that were repeated and 10 filler items that were shown only once. There was an average delay of 10 trials between the 1^st^ and 2^nd^ presentation of each face (range 8–12 trials) which, depending upon the participants’ reaction times, gave a time delay of ∼60 s.

The response cue consisted of the words ‘New-Old’ or ‘Old-New’, randomly varied between trials. Participants were required to press either the left or the right hand side button on a response pad to correspond with the side on which the ‘New’ or ‘Old’ cue was presented. Randomization of the response cue was designed to avoid motor responses while the memory stimulus was still visible because participants would not know what the correct response would be until after the stimulus had been removed. The inter-trial interval varied between 1000 ms and 2000 ms and consisted of a blank screen.

#### Materials and equipment

Twenty-eight electrodes were positioned on the scalp using an ECI electrode cap with electrodes placed according to the International 10–20 system with an additional nine electrodes: Oz, FC5/6, CP1/2, CP5/6 PO1/2. In addition, the horizontal electro-oculogram (EOG) was recorded from the external canthus of each eye and the vertical EOG was recorded from the supra- to suborbit of the left eye. Electrode impedances were all under 5 kΩ. EEG and EOG were amplified using a 32 channel Neuroscan Synapse-II System. Signal bandpass was 0.1–100 Hz and the digital sampling frequency was 500 Hz. Reference was to the left ear and converted to average reference offline.

#### Signal preparation

EEG was divided into segments from −2000 to +2000 ms where zero was defined as the time of stimulus onset. All trials in which the participant gave a correct response (referred to as ‘true new’ and ‘true old’ for the correct identification of first presentation and repeats respectively) and which did not include values outside of the −120 µ to +120 µV range were included in the analysis. ERPs were calculated for each condition separately from the mean of all baseline corrected (−200 ms to 0) trials and the resulting average was smoothed using a zero-phase FIR 20 Hz low-pass filter. ERD/ERS was calculated from the mean amplitude envelope derived from the Hilbert transform of the bandpass filtered trials. Bandpass filtering was by zero-phase FIR filter in the delta (0.1–3.9 Hz), theta (4–7.9 Hz) alpha (8–12.9 Hz), beta1 (13–19.9 Hz), beta2 (20–29.9 Hz) and gamma (30–48 Hz) frequency ranges. ERD/ERS were converted to a percentage of the mean amplitude recorded in the given frequency range in the −500 to 0 ms interval. The power spectrum of the pre- and post-stimulus intervals was estimated using FFT following Welch’s method with a Hanning window. The pre-stimulus segment ran from −1024 ms to −1 ms and the post-stimulus segment from +1 ms to 1024 ms, giving 512 data points in each case resulting in a frequency resolution of 0.98 Hz.

EMD was calculated following the algorithm described in Figure 3 and six IMFs were extracted from each trial. Estimates of the evoked, induced, phase and frequency time series of each IMF were calculated as follows. The evoked component of the signal was estimated from the mean of each IMF averaged for the ‘true new’ and ‘true old’ conditions separately. The Hilbert transform was used to calculate the amplitude envelope amplitude and instantaneous phase of each IMF. The induced component was the mean amplitude envelope. Phase synchrony was measured using the Phase-locking Value [32] calculated from the mean phase vector at each time point averaged across trials. The frequency component was calculated from the 1^st^ derivative (i.e. the gradient) of the unwrapped phase averaged across trials. Numerical differentiation is problematic because it can greatly amplify the noise in the data and several different methods were tried but one based on the continuous wavelet transform significantly outperformed other methods [33] and this is the one used here. Data were averaged by ordinal IMF number such that the first IMFs from each epoch were averaged for each individual to form the mean evoked signal for IMF1, data from the second IMFs were averaged to form IMF2 and so on down to IMF6. The amplitude, phase synchrony and frequency were averaged in the same way. The grand average data for the evoked signal, amplitude, phase synchrony and frequency were similarly obtained by averaging by ordinal IMF number across participants.

#### Statistical analysis

Event-related changes in the EEG were analyzed using Partial Least Squares analysis (PLS)[34]. PLS is a method for determining whether the values of a multivariate dataset are systematically affected by experimental manipulation, in this case, the comparison of the EEG responses to ‘True New’ and ‘True Old’ stimuli. PLS is somewhat like a combination of Principal Components Analysis and multiple regression in that its aim is to identify a latent variable (i.e. a linear combination of the data) that maximally covaries (in a partial least-squares sense) with each component of the experimental design. It does this by performing singular value decomposition (SVD) of the cross-block covariance matrix, which is the matrix containing the covariances between the design matrix and the dependent measures. The SVD generates singular values for each latent variable which indicate the relative importance of each component of the experimental design by showing the proportion of the cross-block covariance accounted for. The statistical significance of each latent variable cannot be calculated analytically so permutation testing is used instead. The rationale for this is that if the experimental manipulation has an effect, the singular values obtained from the dataset grouped according to the actual experimental conditions should be larger than those obtained from an arbitrary grouping of the same data. To test this, PLS is performed a large number of times on permutations of the data in which the allocation of experimental condition for each participant is randomly re-ordered each time (in this case by randomly swapping data between the ‘True New’ and ‘True Old’ conditions). The statistical significance of each latent variable is estimated from the proportion of permuted singular values that are larger than the singular values obtained from the un-permuted data.

Once a latent variable is found to be statistically significant, one can proceed to identify those elements of the data that contribute most to the differences seen. This is done using weightings from the SVD, known as saliences, which show the contribution of each dependent variable (i.e. each time point for each channel included in the analysis), to the latent variable in question. The standard error of each salience can be estimated from bootstrap re-sampling of participants with replacement, keeping the experimental conditions fixed. The reliability of the salience is derived from the ratio of the salience to the bootstrap standard error of the salience which provides a metric equivalent to a z-score. The statistical significance of the PLS was determined using permutation testing with 1000 permutations, and the reliability of the saliences (i.e. where and when the Latent Variable was significantly greater than zero) was established using bootstrapping with 1000 re-samplings.

## Results

Before determining whether there was any evidence for a post-stimulus frequency change in the EEG, it is necessary to demonstrate that the expected changes in the EEG amplitude spectrum, ERD/ERS and ERP were observed in this sample.

### Changes in the EEG Power Spectrum Density

**Figure 5:**
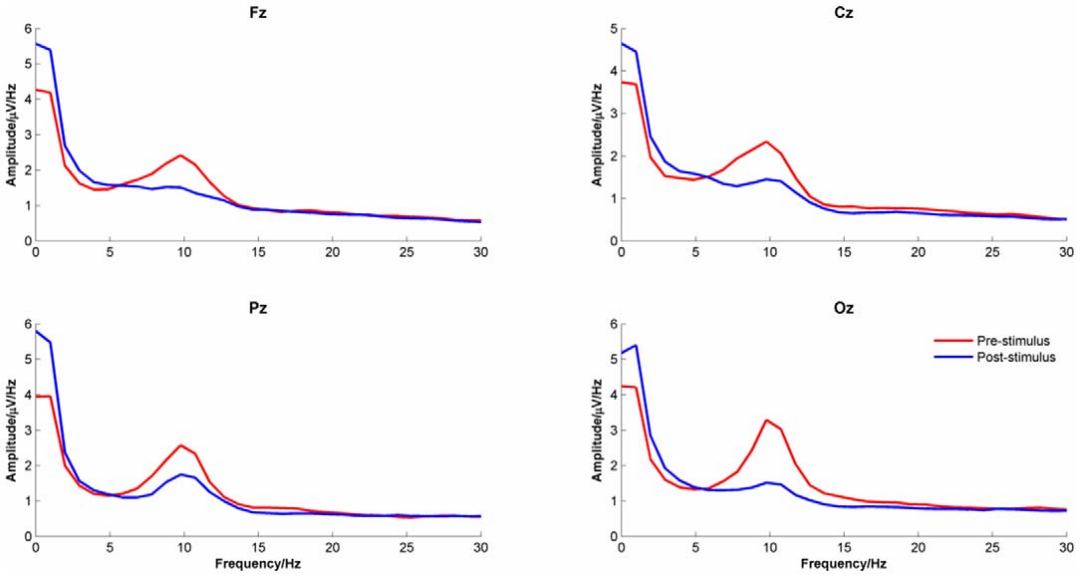
EEG Power Density Spectrum for the pre- and post-stimulus time intervals. Data were averaged across the midline channels (Fz, Cz, Pz & Oz) and conditions (‘true new’ vs. ‘true old’).

Figure 5 shows the EEG power spectrum density in the pre-stimulus and post-stimulus periods for the four midline electrode channels. As expected, at each channel there was a clear reduction in alpha power from the pre-stimulus to the post-stimulus periods.

### Event-related Potentials

**Figure 6:**
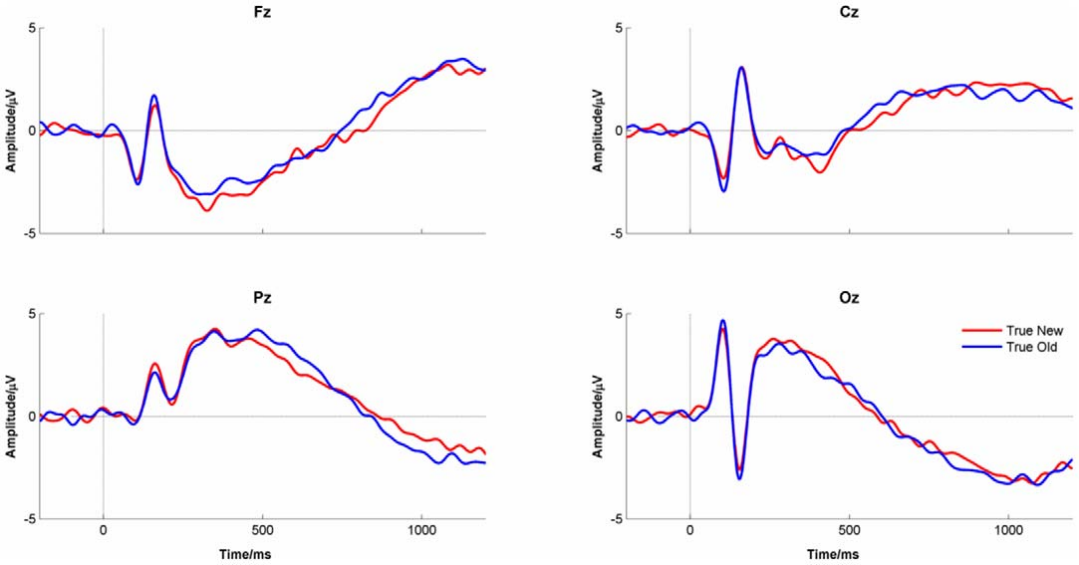
The ERPs for ‘true new’ and ‘true old’ faces for the midline electrode channels.

Figure 6 shows the ERP to ‘true new’ and ‘true old’ faces at the 4 midline electrode channels. Note the phase inversion between Oz and Cz shown by the reversal of polarity of the first two peaks in the ERP. There was a significant difference in the ERPs between conditions (PLS permutation, p<.002). Bootstrap sampling indicated that the differences occurred at two time points. First, the ERP was significantly more positive to ‘true old’ faces than to ‘true new’ faces around 400 ms at Fz and Cz. Second, there was a more prolonged increased positivity to ‘true old’ faces from 500 ms onwards that was maximal at Pz. This findings are consistent with previous studies of recognition memory using ERPs [35].

### Event-related Desynchronization/Synchronization

**Figure 7:**
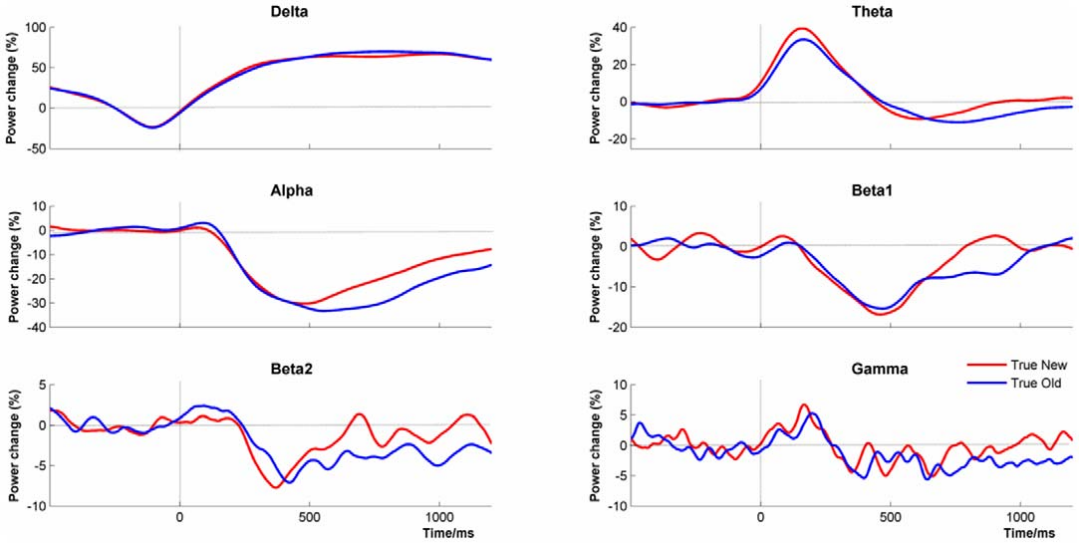
Event-related (De) Synchronization Changes by Frequency Band. Data were averaged in the delta, theta, alpha, beta1, beta2 and gamma frequency bands by condition (‘true new’ vs. ‘true old’) averaged across channels (Fz, Cz, Pz & Oz).

ERD/ERS changes are shown in Figure 7. As the shape of the amplitude changes did not vary much between electrode channels, the data shown are the mean values for all four midline channels. In Delta, the EEG amplitude was not stable in the pre-stimulus period as there was a steady decline in amplitude from −1000 ms to −100 ms. This trend in the baseline amplitude was due to a Contingent Negative Variation (CNV) caused by having a predictable time interval between the stimulus cue and the stimulus onset (Figure 4). Post stimulus, Delta amplitude increased rapidly to above the pre-stimulus level. Similarly, in theta, there was a clear increase in amplitude compared to baseline reaching a maximum around 150 ms (i.e. synchronization) before returning to baseline levels. All higher frequencies showed a reduction in EEG amplitude compared with baseline (i.e. desynchronization).

There was a significant difference between conditions in Delta (PLS p<.001) such that there was higher amplitude to ‘true old’ faces at Pz from 550–1100 ms. For alpha, there was greater desynchronization to ‘true old’ faces at Fz and Cz from 750–1100 ms (PLS, p<.047). In beta1, there was again greater synchronization at Cz, Pz and Oz from 850–1000 ms (PLS, p<.033). There were no significant differences between conditions at other frequency bands.

### Intrinsic Mode Functions

The first 6 IMFs are reported here whose mean frequencies in the baseline period were 78.8, 35.3, 15.5, 7.7, 3.7 and 1.7 Hz respectively.

#### Frequency

**Figure 8:**
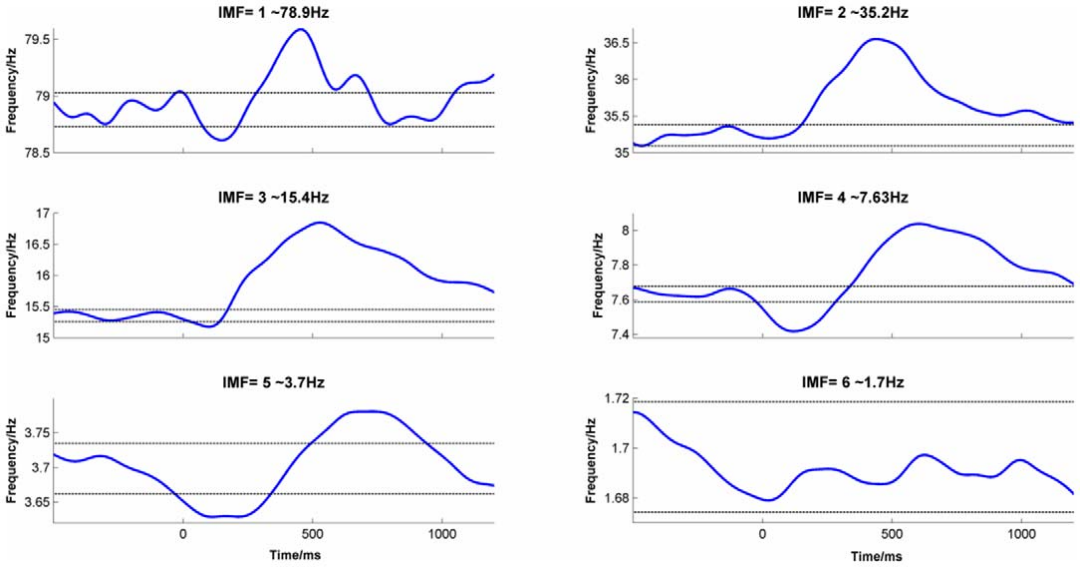
Change in frequency over time for the first 6 IMFs. Data were averaged across channels (Fz, Cz, Pz & Oz) and conditions (‘true new’ vs. ‘true old’). The dotted lines indicate 95% C.I. of the mean frequency in the −500 ms to 0 ms baseline period.

Figure 8 shows the mean Frequency by time for IMFs 1 to 6 averaged across conditions and channels with the mean baseline frequency and 95% C.I. As predicted, there was a change in frequency in the post-stimulus period for most IMFs. The one exception was IMF 6 and in this case the frequency had not been stable in the baseline period. Of the 5 IMFs that showed a post-stimulus frequency change, 4 showed an initial drop in frequency followed by a rebound increase in frequency later on. Only in the case of IMF 2, was the initial drop in frequency not seen, although there was a clear increase in frequency around 450 ms.

#### Amplitude, evoked, phase synchrony and frequency

**Figure 9:**
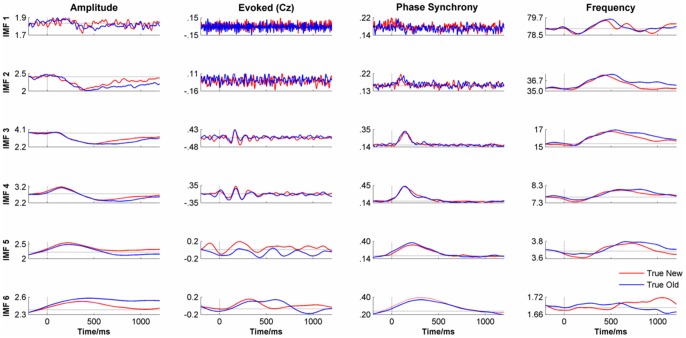
The time course of amplitude, evoked response, phase synchrony and frequency for IMFs 1–6. Data were averaged by condition (‘true new’ vs. ‘true old’) and across channels (Fz, Cz, Pz & Oz) except for the Evoked signal which shows the data for Cz only.

Figure 9 shows the change in amplitude, evoked, phase synchrony and frequency by time and experimental condition of the first 6 IMFs. The results of the PLS analysis by condition are reported in Table 1. Only amplitude showed reliable differences across the frequency range (IMFs 2–5) and evoked showed reliable differences at low frequency, (IMFs 5 & 6).

**Table 1:** Showing the Results of PLS Analysis Comparing ‘true new’ and ‘true old’ stimuli for IMFs 1–6 for Amplitude, Evoked, Phase and Frequency.

IMF	BaselineFrequency/Hz		Amplitude	Evoked	Phase	Frequency
	Mean	s.d.				
**1**	78.8	7.7	.430	.260	**0.01**	.840
**2**	35.3	6.0	**.010**	.560	.220	**.001**
**3**	15.5	3.7	**.001**	.250	.490	.090
**4**	7.7	2.0	**.001**	.700	**.010**	.650
**5**	3.7	1.2	**.050**	**.010**	.280	.850
**6**	1.7	0.7	.170	**.001**	.170	.230

Bold lettering indicates that there was a significant difference between ‘true new’ and ‘true old’ stimuli by the PLS permutation test.

One striking feature of the IMF responses is that the shape of the time course in each response type (amplitude, evoked, phase synchrony and frequency) was similar across all IMFs. This self-similarity reveals a fractal organisation in the IMF response to a stimulus across a broad range of frequencies. The main difference between IMFs was that the response increased in latency and was stretched over a longer period as IMF number increased. For example, in the case of Amplitude, each IMF showed a small, short-duration increase in amplitude followed by a deeper and more prolonged decrease, the main difference being that the latency of these events increased with each IMF. A similar pattern was seen for Frequency except that in this case there was a decrease in frequency preceding an increase. For the evoked response, there was no clear signal in IMFs 1–2 but each of the others show a clear ‘W’-shaped response whose length increased with IMF number. Although there was no clear synchrony in IMF 1 for phase synchrony, for all other IMFs there was a noticeable peak in phase synchrony post-stimulus. The latency and spread of this peak both increased with IMF number.

#### Correlations between the IMF responses

Casual inspection of the time courses of the IMF amplitude and frequency responses suggested that they might be inversely related to each other. To test this, the zero-lagged correlation coefficients of the time courses (−200 ms to +1000 ms) were calculated between the amplitude, evoked, phase-synchrony and frequency responses for each IMF. In the case of the evoked response, the Hilbert amplitude envelope of the response was used rather than the evoked response. The results of this analysis are shown in Table 2. The results indicated that for amplitude, evoked and phase-synchrony responses, the correlations were all positive, most strongly so, with 14 out of 18 exceeding a value of +0.8. In contrast, the correlations with frequency, were all negative, mostly strongly so, with 7 out of 18 correlations being less than −.80.

**Table 2:** Showing the correlations between the Amplitude, Evoked (Amplitude Modulation), Phase Synchrony and Frequency of the IMFs in the time range 0 to 1200 ms.

IMF	Amplitude &Evoked	Amplitude &Phase	Amplitude &Frequency	Evoked &Phase	Evoked &Frequency	Phase &Frequency
**1**	.43	.19	−.51	.72	−.24	−.07
**2**	.83	.45	−.81	.79	−.57	−.20
**3**	.81	.60	−.94	.94	−.67	−.44
**4**	.94	.91	−.96	.99	−.85	−.82
**5**	.99	.99	−.64	.99	−.61	−.66
**6**	.88	.84	−.56	.99	−.81	−.85

### Discussion of the Empirical Study

The main purpose in reporting these experimental results was to determine whether there was any evidence for a change in frequency in the post-stimulus period. To start, however, it was important to demonstrate that this data set showed the event-related changes that would normally be expected in such a paradigm and that there was nothing anomalous or unusual in the results. The data generated an ERP that was comparable to previous studies investigating recognition memory. There was also a post-stimulus attenuation of the alpha peak in the FFT as would be expected. Finally, the ERD/ERS changes showed that there was synchronization in the theta frequency range and desynchronization at higher frequencies, again very similar to previous findings in this area. Overall, these data show a normal pattern of event-related EEG changes.

The frequency-shift hypothesis predicted that there would be an event-related change in frequency of the EEG, specifically in the frequency responses of the IMFs extracted using EMD. The hypothesis was not directional and did not specify whether the frequency would increase or slow down as either way would permit the underlying oscillations to align. However, it was predicted that whichever direction the frequency change was, the change would reverse shortly after. The rationale for this was that if the oscillations changed frequency in order to synchronize, then they would remain synchronized as long as the frequency change was maintained.

The data confirmed that there was a change in frequency (Figure 8). Specifically, the IMFs slowed down to synchronize and then speeded-up again to shift out of alignment. This is consistent with the phase-shift hypothesis but does not on its own allow us to reject the evoked model. To do that, it is also necessary to show that simulated data generated by the evoked model, does not show a similar shift in frequency. Such simulations will be reported later.

Although EMD decomposes the EEG into multiple IMFs, each with a broadly defined and time-varying frequency range, it should not be assumed that these represent an accurate representation of the latent frequency-band structure of the signal (even if such a thing exists). Rather, like any other blind signal separation method (for example, PCA or ICA), it provides a decomposition of the signal that may be useful or convenient for some purposes whether or not it produces an accurate representation of the deep structure of the signal. For example, in the case of signals consisting of white noise, EMD acts as a dyadic filter bank somewhat akin to wavelet decomposition [36], [37]. In such cases, the IMFs do not represent the underlying structure of the signal, for there is none, but the decomposition may prove useful nevertheless. For example, EMD would reveal time-varying changes in the frequency of the noise as changes over time in the frequency of the IMFs.

It is also worth adding a note of caution about the frequency ranges of the IMFs extracted. Although the mean frequency of IMFs across participants was remarkably consistent, the frequency of individual IMFs varied significantly from epoch to epoch (see Table 1). This means that when the IMFs were aggregated across epochs and across participants, they may not have always aligned correctly. For example, depending upon factors such as the noise in a given epoch, an alpha component (∼10 Hz) might sometimes be extracted as IMF3 and sometimes as IMF4, The aggregated IMFs, therefore, may not be homogeneous in terms of frequency with the consequence that any differences in the event-related response between frequencies would be distributed across more than one IMF. For example, if oscillations in the alpha frequency range showed a particular event-related response, this might be detected as event-related changes in either IMF3 or IMF4 or perhaps both. However, given the distribution of frequencies contributing to each IMF (IMFs 1–3 showed virtually no overlap in frequency and IMFs 3–6 were separated by more than 1 s.d), it is possible that frequency-specific changes might affect adjacent IMFs although it is unlikely that such changes would spread further afield. Frequency-specific event-related changes, therefore, would be unlikely to spread across more than two IMFs and the fact that all IMFs showed the same pattern of event-related responses is difficult to reconcile with the existence of any such events.

**Figure 10:**
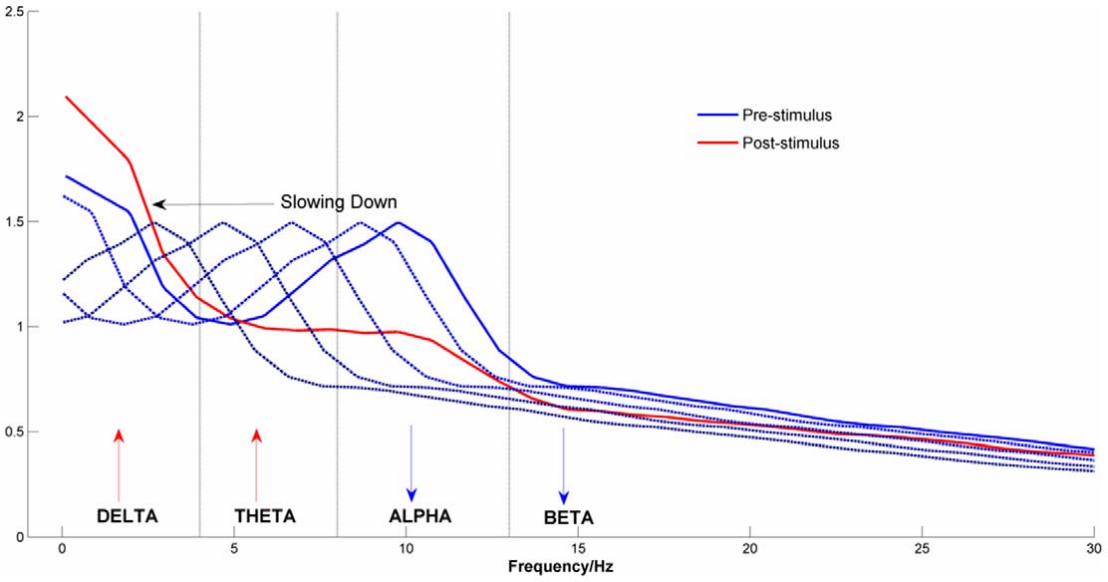
Schematic Representation of the Firefly Model in the Frequency Domain. The blue line indicates the pre-stimulus power density spectrum. The dotted lines indicate the shift in the power density spectrum as the ongoing EEG slows down to synchronize post stimulus for several different degrees of phase disparity. As the size of the frequency shift is determined by the phase disparity, which varies randomly between trials, the average power density spectrum in the post stimulus period will be equivalent to the average of multiple pre-stimulus power-density spectra shifted in frequency (red line).

Perhaps the most unexpected finding was that the time courses of the induced, evoked, phase and frequency of the IMFs were consistently and highly correlated with each other. It is important to recognize that this need not have been so. There is no necessary reason, for example, why the changes in amplitude over time should correlate with changes in frequency and it is easy to conceive of systems where they are independent, for example, AM and FM radio signals. This lack of independence was so striking and unexpected that it requires some explanation.

The first clue to what this explanation might be comes from combining two apparently unrelated observations. First, the EEG synchronized in the post-stimulus period by slowing down. Second, apart from a clear peak in the alpha frequency range, EEG amplitude monotonically diminished with increasing frequency (Figure 5). Combining these two facts suggests that high amplitude oscillations that are in the alpha range in the pre-stimulus period will shift into the theta frequency range post stimulus onset. As power in the pre-stimulus period is greater in the alpha frequency range than in the theta frequency range, this will be detected as an increase in theta post-stimulus (i.e. theta synchronization). In the same way, oscillations in the beta frequency range in the pre-stimulus period will slow into the alpha range post-stimulus. As pre-stimulus beta power is lower than pre-stimulus alpha power, this will appear as an event-related reduction in alpha (i.e. desynchronization). In general, using conventional analyses with fixed frequency bands, and the shape of the pre-stimulus power density spectrum, we should expect to see an increase in theta power post stimulus), but a reduction of power at higher frequencies.

One might expect that this slowing down would simply result in a translation of the FFT spectrum towards lower frequencies, which is not the case as Figure 5 shows. Although there was clear attenuation of the alpha peak and an increase in the delta frequency range, there was no simple shift of the spectrum towards lower frequencies. The simplest explanation for this is that the extent to which the EEG slows down depends upon the phase conditions in the baseline which we can assume to be random. On some trials, the phase change required to synchronize will be small, resulting in a small change in frequency, on other trials it might be large. Averaging the pre-stimulus FFT spectrum over many trials with different shifts in frequency will produce a post-stimulus spectrum in which the alpha peak is ‘smeared’ over a range of lower frequencies. The slowing of oscillations that are in the alpha frequency range pre-stimulus will bring them into the theta range post-stimulus. This conceptualisation is represented schematically in Figure 10. The importance of this conceptualisation is that it provides a candidate mechanism for generating evoked signals that can also explain induced changes. The rest of this paper will be devoted to developing this schematic concept and producing a formal model that can be used to simulate event-related changes in the EEG and which can be tested against observation.

## Models

### Conceptual Outline of the Firefly Model

The essence of the model outlined below is that the ERP is generated by the transient co-ordination of the phases of cortical oscillations across the frequency range. That is, neurones adjust their frequency of firing such that the emergent cortical oscillations become synchronized. Synchronization through frequency adjustment is widely seen in the natural world and one of the best known examples comes from the collective behaviour of certain species of firefly. These fireflies have a natural, preferred rate of emitting light and, left to their own devices, will flash at their preferred frequency. At times, however, they gather in large numbers and when they do, they rapidly synchronize such that they flash in unison. The way in which the fireflies become synchronized has been modelled mathematically [38] and the proposed mechanism is very similar to that outlined below, hence the Firefly model.

**Figure 11:**
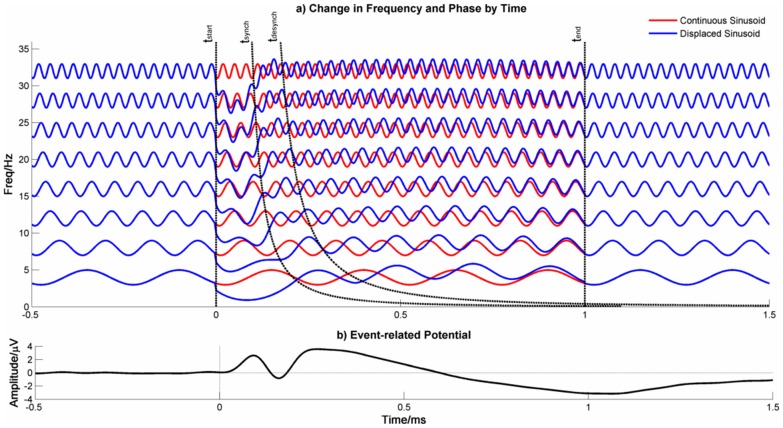
Schematic Representation of the Firefly Model in the Time Domain. Panel a) shows the time course of EEG oscillations across a range of frequencies. The red lines indicate oscillations that are unchanged across the time course whereas the blue lines indicate oscillations that are displaced by the presentation of a stimulus at **t_start_**. The displaced oscillations slow down until they synchronize at **t_synch_**, remain synchronized until **t_desynch_** and then speed up to return to baseline phase by **t_end_**. The synchronization time, **t_synch_**, varies with frequency (black dotted line) such that low frequencies synchronize later than earlier ones. As higher frequencies slow down, they overlap with lower frequencies providing an opportunity for cross-frequency synchronization. Note that the sinusoidal variation on each line indicates that the lines represent oscillations and not that the frequency of the oscillations changes. Frequency changes are represented by variation in the mean value of each sinusoid. Panel b) shows the event-related potential and is sum of the oscillations in panel a).

The Firefly model is outlined schematically in Figure 11. In the pre-stimulus period, there are multiple oscillations, each representing the external field generated by networks of neurones synchronized at a preferred frequency. It should not be assumed, however, that the frequency of oscillations in the local field potential will be the same as the mean frequency of oscillations of the networks of neurons that generate it. In the hippocampus, for example, place cells have been shown to exhibit a higher mean frequency of firing than the theta frequency of the LFP that they produce [39].

**Figure 12:**
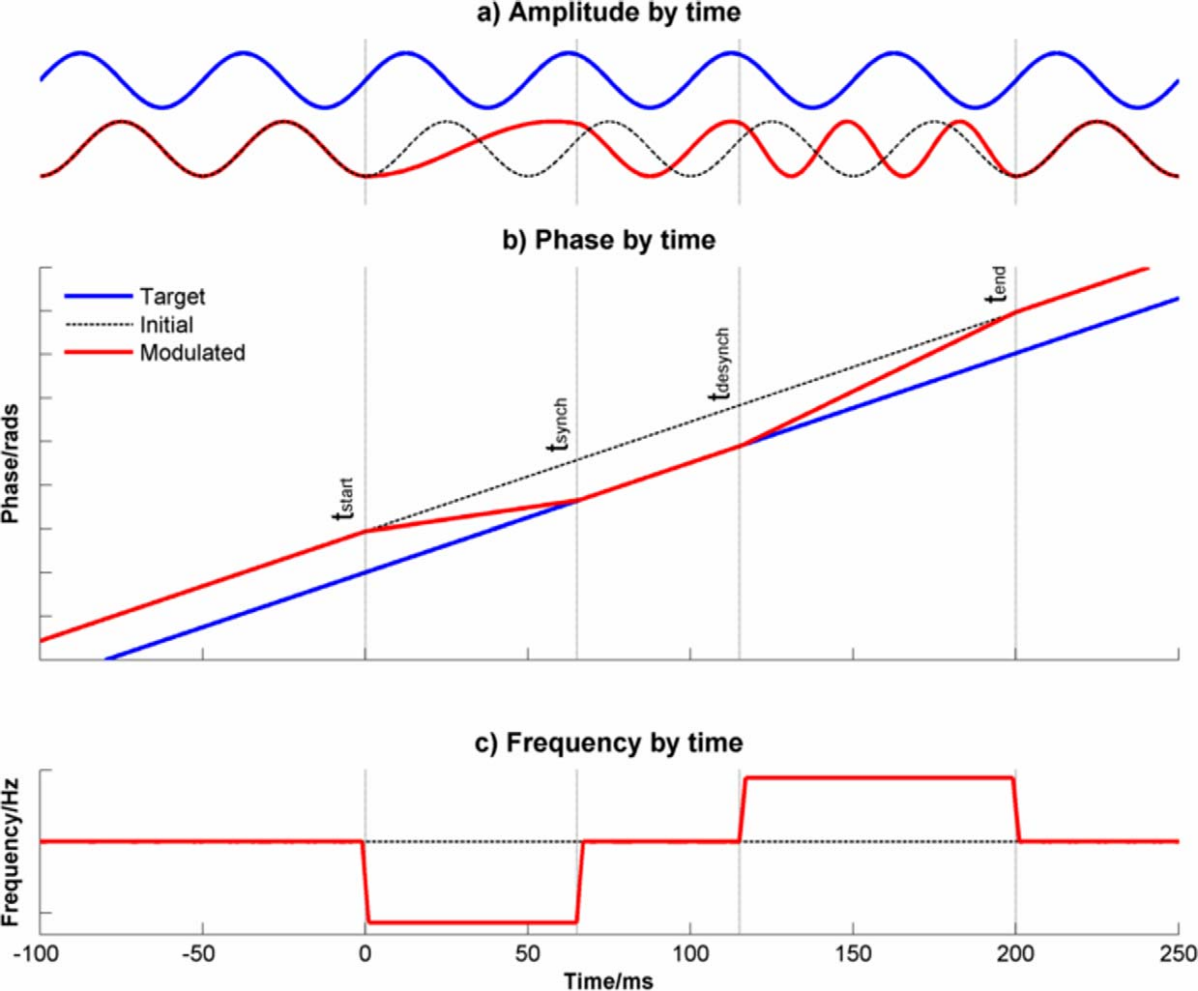
The Firefly Model for a single frequency of oscillation. Panel a) shows the change in amplitude over time. The blue line is the target oscillation and the dotted lines line indicates an oscillation with the same frequency but different phase. The red line indicates an oscillation that at baseline has the same frequency and phase as the dotted black line but whose phase is modulated over time to synchronize and then desynchronize with the target oscillation. At t_start_, the red oscillation slows down and synchronizes with the target oscillation (blue) by t_synch_. From t_synch_ to t_desynch_, the blue and red oscillations remain in phase and from t_desynch_ to t_end_, the red oscillation speeds up to return to its baseline phase and frequency. Panel b) shows the same information in terms of change of unwrapped phase over time. Note the gradient of the lines shows the change of phase over time i.e. frequency. Panel c) shows the same information in terms of change of frequency over time.

At each frequency, there will be multiple networks of neurones oscillating at the same frequency but out of phase with each other. At stimulus onset, these networks adjust their frequency so that they transiently phase-synchronize with each other. In each case, phase synchronization is achieved by slowing the rate of oscillation but the degree of slowing will vary between different networks to an extent determined by the magnitude of the phase shift required to achieve synchronization. Phase synchronization starts at high frequencies and progresses systematically across the frequency range. It is this phase synchronization between these neuronal networks with the same preferred frequency of oscillation, co-ordinated across the frequency range, which produces the ERP. In each case, the short-term slowing is followed by a rebound increase in frequency before returning to the preferred rate. Note that Figure 10 and Figure 11 are equivalent to each other except that one represents the model in the frequency domain (Figure 10) and explains the difference in the Power Spectrum Density pre- and post-stimulus whereas the other represents the model in the time domain (Figure 11). For completeness, Figure 12 shows the model in terms of changes of phase and frequency by time.

### Formalization of the Firefly Model

#### Assumptions of the model

SUMMATION: The EEG can be represented as the sum of many sinusoidal oscillations, each of which represents activity in a neuronal network that oscillates at a preferred frequency.

AMPLITUDE: The amplitude of the oscillations is constant and there is no difference in the overall power of the EEG in the pre-and post-stimulus period. This means that there are no evoked or induced power changes.

PREFERRED FREQUENCY: The frequency of the oscillations may vary over time but each network will have a preferred frequency of oscillation to which they will tend to return.

PHASE ALIGNMENT: phase alignment is achieved by each sinusoid slowing in frequency to reach a target phase at a predetermined time post-stimulus onset. Once phase alignment has been achieved, the oscillation will return to its preferred frequency.

FREQUENCY EQUIVALENCE: event-related changes in the EEG follow the same form for all frequencies. The evidence for this comes from the EMD where the evoked, frequency, amplitude and phase responses were similar across all IMFs.

#### The stages of the firefly model

The essence of the model presented here is that event-related changes in the EEG result from systematic phase alignment across the frequency range achieved by frequency slowing but without any change in amplitude. Figure 12 shows the changes of phase and frequency over time predicted by the model for a single frequency of oscillation. The model assumes that frequency modulation of the EEG can be usefully divided into 5 stages:

**Baseline** (up to **t_start_**). The oscillations are in random phase.

**Synchronization** (**t_start_** to **t_synch_**). The presentation of a stimulus causes the oscillations to shift phase progressively until they reach the target phase at the synchronization time, **t_synch_**.

**Phase-locked** (**t_synch_** to **t_desynch_**). The oscillations remain synchronized until the desynchronization time, **t_desynch_**.

**Desynchronization** (**t_desynch_** to **t_end_**). The process begins to reverse as the oscillations progressively shift back towards their original phase.

**Baseline** (from **t_end_** onwards). The oscillations have returned to their initial phase and frequency.

#### Phase representation of the firefly model

Formally, the model can be represented, in the phase domain (Figure 12b) as:
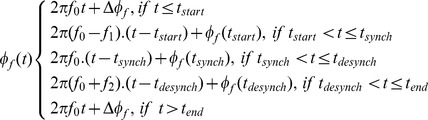
(1)Where ***f_0_***, is initial frequency, ***t***, is time, **Δ** *φ* ***_f_***, is the phase offset for frequency, ***f_0_***, i.e. the difference between the initial and target phases and ***φ*** ***_f_ (t)*** *,* is the phase at time, ***t***.
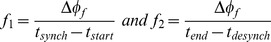



#### Frequency representation of the firefly model

The model can also be represented in the frequency domain (Figure 12c) as follows:
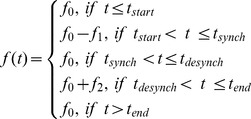
(2)


#### Defining the parameters of the model

The time of stimulus onset, **t_start_**, was defined as 0 ms and all other times are defined in reference to this point. The synchronization time, **t_synch_**, was estimated from empirical data and was defined as the time at which phase-locking index reached its maximum value. Visual inspection of those IMFs that showed a clear peak in the phase-locking index (i.e. IMFs 2 to 6), indicated that **t_synch_** increased monotonically with the period of oscillation of the frequency of the IMF, *T*, (see Figure 9– Phase Synchrony). In fact, **t_synch_**, was proportional to the square root of the period and was well estimated by:
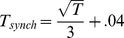
where *T* is measured in seconds and R^2^ = 0.962 and RMSE = .016.

The desynchronization time, **t_desynch_**, was defined as the time at which the oscillations started to return to their baseline phases. The phase-locked period was defined as the time that the phase-locking index remained within 5% of its maximum value and this was measured from the mean phase-locking indices for IMFs 2–6. The relationship between the period of oscillation, *T*, and the desynchronization time, **t_desynch_**, was also proportional to the square root of the period and was well estimated by:
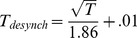
where R^2^ = 0.99 and, RMSE = .004. The end time, **t_end_**, was defined as the time by which the oscillations had returned to their baseline phase. In principle, this could have been estimated from empirical data but in practice, it was difficult to measure so the time by which the IMF frequency had returned to its baseline value was used instead. Inspection of Figure 8 indicated that for most IMFs **t_end_** was >>1.0 s and was not obviously related to frequency. Simulations indicated that this parameter was not critical to the results provided that **t_end-_ t_desynch_** was at least as great as **t_synch-_ t_start_** although longer values gave slightly better results. For this reason, **t_end-_ t_desynch_** was set to 1.5 s for all the simulations reported below.

#### Defining the target oscillation

All phases used in the model were defined in relation to a hypothetical target oscillation which was defined by its phase, **φ_target_**_,_ at a specified time point, **t_target_**, with a separate target oscillation being defined for each frequency. In some ways, the definition of the target oscillation was arbitrary in that it made no difference to the estimation of the power-density spectra or the ERD/S. However, for the ERPs, the definition of the target oscillation was critical in that it defined their shape. If, for example, the phase of the target oscillation, **φ_target_**_,_ varied randomly across frequencies, even though the target and trial oscillations in any given frequency would synchronize, oscillations at different frequencies would synchronize at different phases. The result would be that oscillations at different frequencies would destructively interfere and their sum would tend to zero. The same argument would apply if the target phase, **φ_target_**, **φ** was fixed across frequencies but randomly varied between trials. Clearly, some mechanism for allowing constructive interference was required.

The simplest solution was to define the target oscillation using the same target phase for all frequencies and for all trials and setting the target time as the point of synchronization, i.e., **t_target_** =  **t_synch_**. This guaranteed constructive interference because oscillations of all frequencies would be in phase when they synchronized but this arrangement did not produce an ERP-like waveform. It also had the disadvantage that the target oscillation was not defined until the time at which synchronization was supposed to have occurred which raised the question of how the oscillation ‘knew’ what phase shift to make.

A better solution was found by defining the target oscillation using the same target phase for all frequencies and for all trials but setting the target time as the point of stimulus onset, i.e., **t_target_**_._ =  **t_start_**. This had the advantage of defining the target oscillation at the start of the trial so that the phase offset at each frequency was determined from the start. Its most obvious drawback, however, was that it did not guarantee constructive interference. In fact, it guaranteed that different frequencies would synchronize to different phases at different times and one might reasonably expect that such a pattern would sum to zero and there would be no ERP. However, as the timing of the point of synchronization, **t_synch_**, and the required phase shift both systematically depended upon frequency, the result was a pattern of early synchronization at high frequencies leading to later and later synchronization at lower and lower frequencies. That is, the, the down-chirp pattern of the ERP emerged.

Having defined the target time, **t_target_**, all that was required to define the target oscillation was to specify the target phase, **φ_target_**. The target phase was the same for all frequencies and all trials and could vary from 0 to 2π but within these limits, all values of **φ_target_** produced ERP-like waveforms. The target phase for each electrode channel was found by systematic search and was identified as the value of **φ_target_** that produced the highest correlation between the empirical and simulated ERPs.

#### Simulating a trial of data

Simulated data for each trial consisted of a single sinusoid for each frequency in the range 0.1 Hz to 250 Hz at 0.1 Hz intervals offset from the target oscillation by a random amount, **Δφ_f_**, in the range - π to +π radians. Each sinusoid was phase-modulated so that it became in phase (i.e. synchronized) with the target oscillation by **t_synch_**, remained phase-locked until **t_desynch_** and returned to its initial phase by **t_end_**.

The phase modulation could have been achieved in many ways. For example, following the phase re-setting model, phase could have been instantaneously reset to the target phase at **t_synch_**, but this did not give good results in that it did not generate ERP-like waveforms. Instead, a progressive phase shift was used and, for simplicity, it was assumed that the transition would be linear i.e. (**φ_target_ - φ_start_)/(t_synch_** - **t_start_**). Quadratic and cubic spline transitions were also considered and gave similar results in the simulations suggesting that the choice of transition was not critical. However, as the linear model was simpler and produced marginally better results, these other methods will not be reported here.

Using this procedure, the phase-shifted oscillations for each frequency were generated, *φ_f_(t)* with a random phase offset for each trial. The randomly phase-shifted oscillations were then amplitude modulated so that each frequency was weighted in proportion to its contribution to the overall amplitude of the EEG in the baseline period. The weighting factor for each frequency, *a_f_*, was estimated from the FFT of the pre-baseline period obtained from the empirical data.

A single trial of data, V(*t*), could be calculated from the amplitude modulated sum of the phase-shifted oscillations:
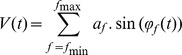



In this way, multiple simulated trials of EEG were generated which could be analysed in exactly the same way as the empirical data to give estimates of the ERP, ERD/S and FFT.

### Comparing Simulated and Empirical Data

The reported simulations consisted of 100 trials each. The degree of similarity between the empirical and simulated results was estimated using Pearson’s correlation co-efficient. For the FFT spectra, the correlation was calculated on the 0–250 Hz range. For the ERP and ERD/S, the time-lagged correlations (±200 ms) were calculated on the 0 to 1000 ms interval.

#### Does Frequency Discriminate Between Evoked and Induced Changes?

**Figure 13:**
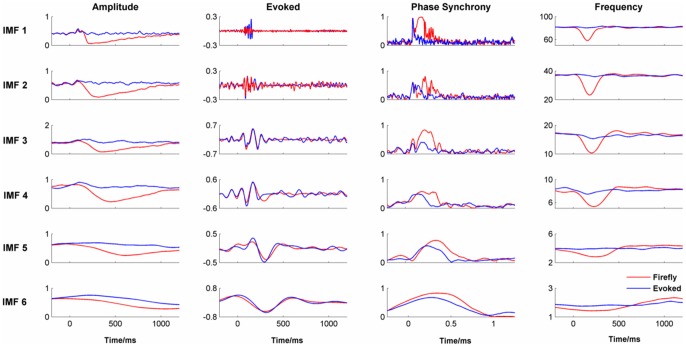
EMD analysis of simulated data generated by the Firefly model and Evoked models.

The first simulation was designed to determine whether the slowing of frequency seen in the empirical data post-stimulus onset could be used to distinguish between the Firefly model and the evoked models. Trials of simulated data were generated using the Firefly model, and averaged to create an ERP. Then, to simulate evoked data, the Firefly model generated ERP was added to trials of simulated data that was generated in exactly the same way as the Firefly model-data except that no phase modulation was used. Trials produced using the Firefly model and evoked models were then subject to EMD and the results are shown in Figure 13. The resulting ERPs were very similar (Figure 13-Evoked). Both evoked and Firefly model generated signals showed an increase in the phase locking index for all IMFs although the PLI was greater for the Firefly model-generated model. However, for both the amplitude and frequency responses, event-related changes were only seen in the Firefly model-generated data.

#### Changes in the power spectrum density

**Figure 14:**
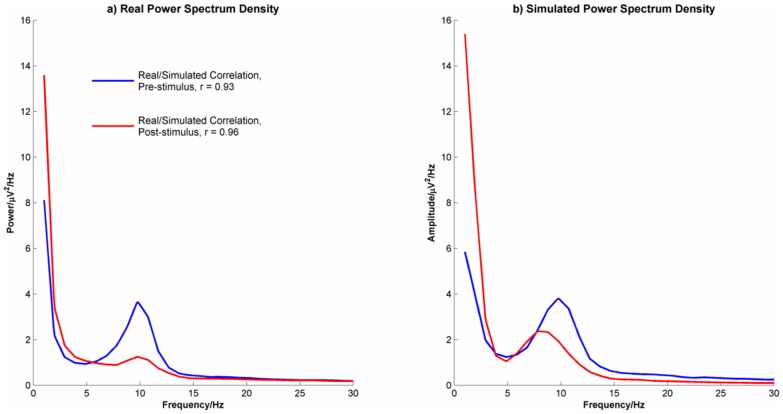
Power Spectrum Density for a) Real Data and b) Firefly simulated data.

The pre- and post-stimulus power spectrum densities, averaged across the four midline electrodes, are shown in Figure 14a). The equivalent Firefly model-simulated data are shown in Figure 14b). The correlations between the empirical and simulated data for the pre- and post-stimulus period were 0.93 and 0.96 respectively suggesting a good match between the two. However, the alpha peak of the simulated data showed a greater shift in frequency and a smaller attenuation of power between the pre-and post-stimulus periods than was seen in the empirical data.

#### Event-related desynchronization/synchronization

**Figure 15:**
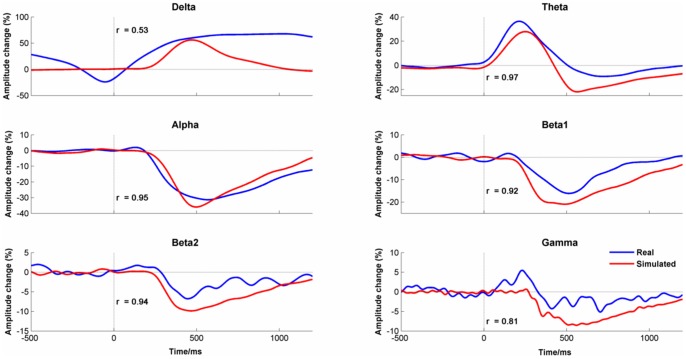
Event-related (de) synchronization changes by frequency band for the real and Firefly simulated data.

Figure 15 shows the empirical and simulated changes in EEG amplitude in the delta, theta, alpha, beta1, beta2 and gamma frequency ranges. Data were averaged across the four midline electrode channels and between conditions. The Firefly model simulated data provided a good match for the theta, alpha and beta1 frequency ranges but was less good in delta, beta2 and gamma. In the case of delta, although the timing of the onset of the increase in power was accurately modelled, the duration of the increase was not. Similarly, but not surprisingly, the CNV seen in the empirical data, shown as a pre-stimulus decrease in delta power, was not accurately reproduced by the Firefly model simulated data. For the higher frequencies, the opposite pattern of discrepancy was seen in that although the shapes of the waveforms were accurately modelled, their timing was not. In an attempt to estimate the discrepancy in timing between the empirical and Firefly model-simulated data, time-lagged correlations between the two were calculated and the maximum correlation and its corresponding lag were identified. In alpha, the maximum correlation was 0.91 with a 0 ms lag suggesting that the timing was accurate in this frequency band. However, the discrepancy in timing increased with frequency and the maximum correlations for beta1, beta2 and gamma were 0.95, 0.84 and 0.81 occurring at lags of 78 ms, 110 ms and 148 ms respectively.

#### Event-related potentials

**Figure 16:**
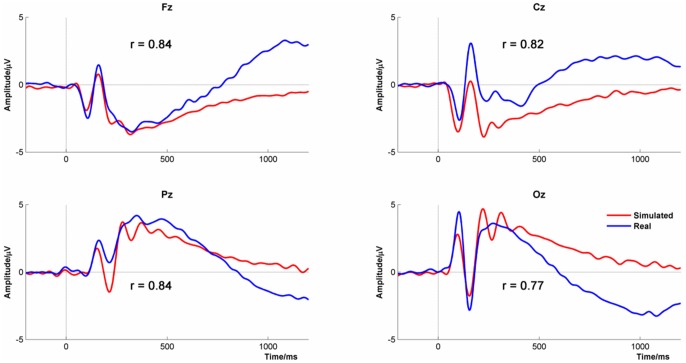
Real and Firefly simulated event-related potentials at channels Fz, Cz, Fz and Oz.

Figure 16 shows the empirical and simulated ERPs for the four midline channels, averaged across experimental conditions. The differences in the simulated ERPs between the channels arose from two sources. First, the ERPs were derived from empirically measured power density spectra that differed between channels. This had an impact on the overall magnitude of the ERP and the relative size of the ERP peaks spectrum. Second, the channels differed in target phase, φ_target_. Target phases for each channel could vary between −180° and +180° and were found by systematic search. The target phases that produced the best reproduction of the real ERPs were +88°, +60°, −96° and −115° for Fz, Cz, Pz and Oz respectively which shows the same ordinal relationship as the azimuth of the polar co-ordinates of the same electrodes in the 10–20 system (45°, 0°, −45° and −90° respectively). Furthermore, the difference in target phase between electrode pairs Fz/Pz and Cz/Oz corresponded very closely with their difference in 10–20 azimuth position being ∼180° in each case. That is, the target phase varied systematically across the scalp.

The empirical and simulated ERPs showed strong similarity with all the correlations exceeding 0.85. There were, however, some differences. For example, the simulated data showed a double peak in the P300 at Pz and Oz which was not seen in the empirical data and a similar difference was present in the phase-inverted P300 at Fz. However, a double peak was seen at Cz in both empirical and simulated data. The other main difference was that the absolute discrepancy in amplitude between the empirical and FM-simulated data increased towards the end of the trial suggesting that there was a low-frequency trend in the empirical data that was not adequately modelled in the simulations.

#### Differences between experimental conditions

For the empirical data, there were no reliable differences in the EMD between the ‘true new’ and ‘true old’ conditions in the evoked, phase or frequency domains. The only reliable differences seen were in the amplitude responses. As the present model explains amplitude differences as a consequence of frequency/phase modulation, the absence of differences in the phase or frequency responses provided no basis for modelling the difference between the experimental conditions.

## General Discussion

I have shown that a simple single-process model can account for many features of event-related changes in the EEG that have previously been interpreted as separate, if not wholly independent phenomena. In particular, the model challenges the distinction that event-related changes in the EEG fall into two distinct categories: evoked and induced. Instead, both types of change can be explained as the consequence of cross-frequency phase modulation with no change in overall EEG power. If this model is correct, the evoked/induced distinction is an artefact that results from analysing data within fixed frequency bands.

The Firefly model belongs within the phase-alignment family of theories and shares important features with many of them, particularly the most fully developed versions such as the Event-Related Phase Reorganization (ERPR) model [40]. All these approaches emphasise the importance of re-organisation of the background EEG and minimize or reject the roles of evoked components. Where the Firefly model scores over all previous phase-re-alignment theories, however, is that it explicitly models the co-ordination of phase-reorganisation across the frequency range and it is this feature that permits it to make specific, quantitative predictions about the full range of event-related changes in the EEG.

Although the match between the empirical and simulated data was good, it was not perfect. Changes in the very lowest frequency range (delta) were not accurately modelled as was shown by the poor correspondence between the empirical and simulated event-related desynchronization in the delta frequency range. Similarly, the increasing deviation over time between the amplitudes of the empirical and simulated event-related potentials suggests that there was a low frequency trend in the empirical data that was not properly accounted for by the model. In part, this might have been due to the experimental design which gave rise to a CNV in the empirical data that was considered to be a nuisance variable and, therefore, made no attempt to model it. The low frequency differences could simply be an artefact resulting from baseline correcting data with a CNV present but it is impossible to exclude other factors.

Our initial model assumed that the event-related changes would start immediately the stimulus was presented but simulations of the ERPs suggested that this was too early. A simple shift of **t_start_** by 80 ms was sufficient to correct this discrepancy and it seems reasonable to assume that at least some of this delay might be accounted for by the time taken for information about a visual stimulus to reach the cortex. Estimates of how long this delay might be are difficult to come by but the earliest visual evoked potential component believed to be cortical in origin occurs around 50 ms [41].

Even after this correction, however, a lag between the empirical and simulated ERD/S data still remained, at least for the higher frequencies. Furthermore, the time lag increased with frequency. Perhaps the most likely explanation for this is that the estimation of **t_synch_**, which was based on an extrapolation of the timing of the peak phase-locking index, was not accurate at the higher frequencies. Alternatively, simply adding some variability to the timing of t**_synch_**, might have had a similar effect. Adding some latency jitter would also tend to eliminate the double ERP peaks in the simulated P300.

A more important discrepancy between the empirical and simulated data was in the magnitude of the frequency shift. The frequency shift in the simulated data was much larger than that seen in the empirical data. Conversely, the amplitude shift was too small. On first consideration, this might seem to be a fatal flaw in the model but the simulations assumed that all the power in the EEG responded to the stimulus event in the way predicted by the model and this may not be realistic. By permitting only a proportion of the EEG to respond, the overall change in frequency dropped rapidly and when the proportion approached ∼50%, the frequency changes approached those seen in the empirical data. Other features of the model were not significantly affected by this change.

With sufficient degrees of freedom, simulations can always be made to match empirical data. A key strength of the present model is that there was only one free parameter, the target phase. Some parameters were defined by the recording equipment (e.g. the frequency range), others by the experimental set-up (e.g. **t_start_**). Of the remainder, some were not critical, at least within quite a broadly defined range of values critical (e.g. **t_end_**), and those that were could be estimated by direct measurement (e.g. **t_synch_**). Only the target phase needed to be determined by systematic search. In a phase-resetting model, the target phase is the phase to which the oscillations are abruptly set by the presentation of the stimulus at **t_start_**. In the present model, the target phase was defined at **t_start_** but the synchronization occurred later at **t_synch_**, the latency of which depended upon frequency. The result was that although the target phases were the same across all frequencies at **t_start_**, each frequency synchronized at a different phase at **t_synch_**. The value of the target phase was not critical for changes in the power spectrum density or the non-phase-locked signal, provided that it was the same for all frequencies. It was critical, however, for the phase-locked changes, as it defined their phase structure which was the primary reason why the event-related potentials differed between channels. The target phases, however, were not random but were found to vary systematically across the scalp in a way consistent with the idea that event-related changes in the EEG behave like a travelling wave across the cortex [17]–[19].

ERPs consist of various peaks and troughs (components) that are differentially responsive to specific aspects of the experimental paradigm such as the nature of the stimuli used and the type of cognitive processing required and any good model should be able to account for these variations. Although the Firefly model provides a good account of the shape of the ERPs reported in the present study, it remains to be seen whether the model can generalise to other paradigms. In principle, however, small changes in the synchronization times across the frequency range should be able to mimic a wide variety of ERP forms. However, it must be acknowledged that one of the main limitations of the Firefly in the present context was that it was unable to provide an explanation for why event-related potentials to faces shown for the first time (New Faces) differed from those that were repeated (Old Faces). It might be expected that there would be systematic differences in the key parameters of the model, such as **t_synch_**. This might be because the differences in ERPs between the conditions were relatively subtle in this case and it may be possible in future, with more accurate measurement of the parameters of the model, to account for differences between experimental conditions. Alternatively, it might be the case that there are real amplitude differences between the two conditions that are not included in the Firefly model but which might be accounted for by some other mechanism, such as the AAM model [21], [22]. Notwithstanding this, the Firefly model alone can account for most of the event-related changes seen, including the peaks and troughs of the ERP which are the event-related changes that are most commonly treated as evoked features.

One of the intriguing aspects of the Firefly model is that it provides a possible mechanism for transferring information between frequencies. Oscillations can only synchronize when their frequencies are in the ratio ***m:n*** when ***m*** and ***n*** are integers. It has been claimed that the mean frequencies of adjacent bands in the resting state are in the ratio ***1:φ***, where ***φ*** is the golden ratio (∼1.618) [7], [8]. It can be shown that a ratio of ***1:φ*** minimizes the probability that peaks or troughs in the oscillations from adjacent frequency bands will coincide, thereby reducing the chance of coincidental synchronization and preventing cross-frequency interference. This means that neuronal networks oscillating at these frequencies will be informationally isolated from each other [7]–[9]. The resting state, therefore, represents a condition in which communication between frequency bands is at its lowest possible level and this makes it ideally prepared to receive and process new sensory input. It is this transition from resting state to the processing of sensory input that the Firefly model attempts to describe.

The modulation of frequencies in the Firefly model enables synchronization between neuronal networks with the same preferred frequency of oscillation but it also permits information exchange between networks with different preferred frequencies. This is because the slowing down in frequency allows networks with different preferred rates of oscillation to overlap in frequency. So, instead of adjacent frequency bands oscillating with ratios of ***1:φ***, the higher frequency slows down so that the ratio becomes ***1:1*** and synchronization can occur. Similarly, the post-synchronization speeding up of frequency might permit information to flow in the reverse direction from neuronal networks with low preferred frequencies of oscillation to higher ones. This is outlined schematically in Figure 11. In the pre-stimulus period, there are multiple oscillations, each representing the external field generated by a neuronal networks synchronized at their preferred frequency. At stimulus onset, each network of neurones slows in frequency and transiently phase-synchronizes with networks of neurones that have preferred rates of oscillation that are lower. These in turn slow down to phase-synchronize with networks with lower preferred frequencies and so on. The slowing starts at high frequencies and progresses systematically across the frequency range. In each case, the short-term slowing is followed by a rebound increase in frequency before returning to the preferred rate.

This idea of cross-frequency information transfer also explains why the oscillations slow down to synchronize rather than speed up. If, as has previously been suggested frequency is inversely proportional to scale [42], then the highest frequencies represent the most localised information processing i.e. the von Stein-Sarnthein hypothesis. As information processing over localised networks is likely to be faster than information processing over widely dispersed networks, high frequency synchronization will occur before lower frequency synchronization. It makes sense, therefore, to pass information downwards through the frequency range instead of upwards, from the small scale to the large and this can only be achieved by slowing the frequency of the oscillations.

The Firefly model treats oscillations of all frequencies in the same way but, as noted in the introduction, there is good reason for thinking that there are at least 8 frequency bands in the 0–100 Hz range. Part of the evidence for these bands comes from the known differential response of different frequencies e.g. theta synchronization and alpha desynchronization. As I have shown, these observations can be accounted for by the Firefly model in quite a different way. This is not to say that the Firefly model is incompatible with the existence of frequency bands. Indeed, the Firefly model provides a possible explanation as to why, with the exception of alpha, there are no distinct frequency peaks in the resting state EEG average power spectrum. The reason is that although neuronal networks may have a preferred frequency of oscillation, this frequency is not rigidly fixed but is able to vary over time. Averaging the FFT over any prolonged period will result in a smooth power spectrum similar to that seen in Figure 5.

The Firefly Model is presented here as a formal model of event-related oscillations in the cerebral cortex with sparse discussion of how it might be implemented at the neural level. The primary reason for this is that the data used for the development of the model (i.e. scalp-recorded EEG), provide very little insight into physiological mechanisms involved. Nevertheless, even though the neural basis of the model is unspecified, there is nothing in the Firefly model that is inherently biologically implausible. The Firefly model, after all, was named after a natural phenomenon (i.e. the synchronized emission of light by large numbers of Fireflies) and the synchronization of hand clapping by applauding audiences provides another example of what is essentially the same mechanism [43].

There are two key elements in the Firefly model i) event-related phase/frequency modulation of the ongoing EEG leading to synchronization and ii) systematic variation of the timing of that synchronization across the frequency range. With regard to the first element of the model, modulation of the phase of neuronal firing in response to environmental signals is a well established phenomenon in the mammalian central nervous system. Probably the best-known example of this is type of phenomenon is hippocampal place cell phase precession [44]. Place cells are neurons in the hippocampus whose activity increases as an animal moves through a specific spatial location [45]. The dominant local field potential (LFP) in the hippocampus shows a characteristic theta oscillation and place cells have a preference to discharge at a specific phase of this rhythm. However, the preferred phase changes systematically as the animal moves in space with the results that the animal’s movement modulates the preferred phase (and therefore, frequency) of neuronal firing (i.e. theta phase precession). Event-related phase modulation of individual neurones controls the degree of synchronization across a network of neurons and it is this synchronization that contributes to the LFP (although the relationship between the two is complex [39] and not always well understood). The EEG is simply the weighted sum of LFPs from a contiguous volume of cortex. For the firefly model, the event-related phenomenon is the presentation of a stimulus rather than movement in space, but otherwise the parallel with theta phase precession is quite close.

The second element of the Firefly model, that there is systematic variation in the timing of synchronization across the frequency range, arises quite naturally from the von Stein-Sarnthein hypothesis [42]. If, as is proposed, the oscillatory frequency is proportional to the size of the neuronal assembly involved, then high frequency oscillations will be used to co-ordinate highly localised networks of neurones and lower frequency oscillations will co-ordinate more dispersed ones. If it is reasonable to assume that larger, more dispersed neuronal assemblies will take longer to synchronize than smaller more localised assemblies, then high frequency oscillations will synchronize earlier than low frequency ones which is exactly what the Firefly model proposes. In short, the Firefly can be readily implemented by neural processes that are known to exist in the mammalian cortex.

If the Firefly model is correct, then it has important ramifications for the analysis and interpretation of event-related changes in the EEG. The widely held view that the peaks and troughs of ERPs represent clearly identifiable ‘components’ with specific functions and sources may be overly simplistic. Although this idea works reasonably well for early sensory ERP components, it seems to fit less well for later cognitive ones and specific sources for these have often proved elusive (see for example [46]). One possible explanation for this is provided by the von Stein-Sarnthein hypothesis. From this perspective, early ERP components which, consist of synchronized high frequency oscillations, reflect activity in small well-localised networks of neurones whereas later components, made up of synchronized low frequency oscillations, indicate activity across much more widely dispersed networks. It might be more appropriate, therefore, to think of an ERP as an event-related travelling wave that starts in one or more narrowly defined locations and which, over the course of a few hundred milliseconds, spreads out to incorporate more and more of the cortex, reducing in frequency as it does so. From this perspective, the goal of identifying the sources or ERP peaks, particularly long latency ones, may prove illusory. Furthermore, if the Firefly model is correct, studying induced and evoked changes as different and independent phenomena is no longer defensible.

The Firefly model presents both challenges and opportunities. The challenges lie in the realm of signal analysis for if the Firefly model is correct, then many of our most popular analytical tools are not fit for purpose as they are predicated on the idea of fixed frequency bands. EMD does not share this assumption and shows much promise but there is currently very little experience of using the technique with EEG so its limitations in this context are not well understood. The opportunities stem from the insights that the Firefly model offers about the nature of event-related changes in the EEG. These insights may prove to be a useful a guide in helping us move from a focus on the surface features of the ERP, such as the amplitude and latency of peaks, towards a study of the true deep structure of event-related changes in the EEG.
